# Supplementary material for: A multiple risk factor program is associated with decreased risk of cardiovascular disease in 70-year-olds: A cohort study from Sweden
Source: PLoS Med. 2020 Jun 11;17(6):e1003135. doi: 10.1371/journal.pmed.1003135 (PMC7289341; doi:10.1371/journal.pmed.1003135)
Supplement: S1 Appendix — (DOCX) [file pmed.1003135.s002.docx]

**S1 Appendix.** Description of the prevention program given within the Healthy Ageing Initiative.

**First visit**

The first visit at the clinic starts with a research nurse who provides participants with general information about the project in Swedish. Participants then answer a comprehensive questionnaire in Swedish with themes such as lifestyle, mood, cognitive function, nutrition, physical activity, health behavior and risk factors for CVD. In addition, the risk of CVD is evaluated based on objective tests, with a written protocol to standardize the procedures of all tests. The tests include measurement of weight and height to estimate body mass index (kg/m^2^), using a digital scale (SECA, GmbH, Hamburg, Germany) and a wall-mounted stadiometer. Systolic and diastolic blood pressure (mmHg) are measured on the right upper arm in a seated position after at least 10 minutes rest using an automatic procedure (OMRON global, Kyoto, Japan). Waist circumference is measured below the last rib, and hip circumference is measured at the widest part of the gluteus maximus using a non-elastic measuring tape. Capillary fasting blood glucose is analyzed using the HemoCue 201 RT system (Radiometer Medical ApS, Denmark), and fasting blood lipids are analyzed at the Department of Clinical Chemistry, Umeå University Hospital. Total, gynoid and android fat mass are measured using dual-energy X-ray absorptiometry (Lunar iDXA; GE Healthcare Lunar, Madison, WI, USA). During testing participants receive continuous information about their results, including motivational support regarding potential lifestyle changes. The visit takes about 3 hours. Afterwards, the participants return home with a hip-mounted triaxial accelerometer (GT3X+; Actigraph, Pensacola, FL, USA) that measures their physical activity during one week, with a second visit scheduled with the same research nurse at the end of the week.

**Second visit**

During the second visit, the research nurse and the participant discuss the test results further, including the results from the objective physical activity measurement. As such, these test results form the basis for a motivational interview in Swedish [1], where the research nurse guides the participant through themes such as healthier diet, weight loss, smoking cessation, and increased physical activity. The impact of the participants´ present diet on blood glucose, triglycerides, HDL and LDL cholesterol levels are explained and reflected up on. Participants are encouraged to increase HDL to >1.0 mmol/L and to lower triglycerides to < 1.7 mmol/L, preferably by increasing physical activity and making dietary changes. General advice is given to reduce saturated fat, fast carbohydrates, alcohol, white bread, and red meat and to increase fish intake, vegetables, long acting carbohydrates and polyunsaturated fat (with examples of food rich in these nutrients). The research nurse further presents, and together with the participant reflect upon, daily graphs of physical activity from the 7-day accelerometer measurement preceding the second visit. Participants are encouraged to increase their physical activity based on the output from the 7-day accelerometer measurement and the guidelines issued by the World Health Organization, recommending at least 150 min of moderate-intensity physical activity (or 75 min of vigorous physical activity) per week [2].

With respect to triglycerides and LDL, participants are recommended to consult their general practitioner if triglyceride levels ≥2 mmol/L and/or LDL ≥4 mmol/L. Blood glucose ≥6,1 mmol/L during the first visit is considered to be increased, and is subsequently measured again at the second visit. Participants with blood glucose values ≥7,0 mmol/L at both the first and second visit are encouraged to contact their general practitioner. Participants with blood glucose values ≥6,1 mmol/L at first and second visit, are encouraged to make lifestyle changes and contact their general practitioner for a follow up after 3 months. With respect to blood pressure, a mean blood pressure >135/85 at the first visit is considered increased and is measured again at the second visit. Participants without risk factors (diabetes, history of myocardial infarction, stroke, kidney disease) with increased mean blood pressure >145/85mm Hg but <180/100 mm Hg at the first visit, are encouraged to measure their blood pressure at home. This should be performed before the second visit to rule out white coat hypertension. Participants with risk factors (diabetes, history of myocardial infarction, stroke, kidney disease) and mean blood pressure >130/80 mm Hg at both first and second visit, are encouraged to contact their general practitioner. A participant is sent directly to their general practitioner or to the emergency ward if the mean blood pressure during the first visit is ≥180/100 mm Hg.

**References**

1. Miller WR, Rose GS. Toward a theory of motivational interviewing. The American psychologist. 2009;64(6):527-37. doi: 10.1037/a0016830. PubMed PMID: 19739882; PubMed Central PMCID: PMC2759607.

2. Global recommendations on physical activity for health. Geneva: World Health Organization. 2010.
