# Supplementary material for: A multiple risk factor program is associated with decreased risk of cardiovascular disease in 70-year-olds: A cohort study from Sweden
Source: PLoS Med. 2020 Jun 11;17(6):e1003135. doi: 10.1371/journal.pmed.1003135 (PMC7289341; doi:10.1371/journal.pmed.1003135)
Supplement: S1 Table — (DOCX) [file pmed.1003135.s003.docx]

| **S1 Table** | | |
| --- | --- | --- |
| **Variable** | **Definition** | **Data Source** |
| Date of birth | For privacy reasons, we obtained only controls’ month and year of birth. All dates of birth were therefore assumed to be the 1^st^ of the month | Register of the Total Population (SCB) |
| Umeå Resident | HAI participant or resident of Umeå in the year of 70^th^ birthday | HAI database or Register of the Total Population (SCB) |
| Socioeconomics |  |  |
| Civil status | Married (including registered partner), never married, divorced (including divorced partner), widow/widower (including deceased’s partner) | Register of the Total Population (SCB) |
| Education | Highest level of completed education (primary, secondary, post-secondary) | Register of the Education of the Population (SCB) |
| Disposable income | SEK in the year of 60^th^ birthday. Negative values coded as missing | Income and Taxation Register (SCB) |
| Diagnoses (ICD-10) |  | National Patient Register (NBHW) |
| Prescription medications (ATC) |  | Prescribed Drug Register (NBHW) |
| Date of Death |  | Cause of Death Register (NBHW) |
| SCB, Statistics Sweden; HAI, Healthy Ageing Initiative; NBHW, National Board of Health and Welfare; ICD-10, International Statistical Classification of Diseases and Related Health Problems, 10^th^ Revision; ATC, Anatomical Therapeutic Chemical Classification System; SEK, Swedish krona | | |
